# Description of an Intraoral Lingual Arch Fixation Technique for the Treatment of Mandibular Body Fractures in Dogs and Its Application in Six Patients

**DOI:** 10.3390/ani16182922

**Published:** 2026-09-17

**Authors:** Fidel San Román-llorens, Alejandro Blanco, Fidel San Román, Cristina González, Alberto Climent, Ana Whyte

**Affiliations:** 1 Department of Animal Pathology, Faculty of Veterinary Science, University of Zaragoza, C. Miguel Servet, 177, 50013 Zaragoza, Spain; elbuzondefidel@hotmail.com (F.S.R.-l.); gonzalezpastormc@unizar.es (C.G.); albertocliment90@gmail.com (A.C.); awhyte@unizar.es (A.W.); 2 Hospital Centro Clínico Veterinario de Zaragoza, C. Madre Genoveva Torres Morales, 8, 50006 Zaragoza, Spain; 3 Hospital de Urgencias Veterinarias de la Región de Murcia, C. Chimeneas, 22, Churra, 30110 Murcia, Spain; 4 Department of Animal Medicine and Surgery, Faculty of Veterinary Science, Complutense University of Madrid, Av. Puerta de Hierro, s/n, 28040 Madrid, Spain; fsanroma@ucm.es

**Keywords:** mandibular, fracture, dogs, intraoral fixation

## Abstract

Mandibular fractures are a common injury in dogs and are associated with relatively high complication rates. The main goals of treatment are to provide stable fixation to promote bone healing, preserve the teeth and surrounding anatomical structures, and restore normal dental occlusion. Several treatment techniques have been described, including external skeletal fixation, bone plates and screws, interdental and intraosseous cerclage wiring, and dental splints used alone or in combination with cerclage fixation. However, all have inherent limitations. The objective of this study was to describe a novel intraoral fixation technique and evaluate its clinical application. The technique consists of a lingual arch anchored to the teeth or mandibular bone using cerclage wires and reinforced with bis-acrylic resin. Six dogs presenting with 11 mandibular body fractures, 10 of them opened toward oral cavity and with anatomically unfavorable configurations, were treated. The fractures were located rostral to or involved the alveolus of the first premolar. Ten fractures were open and potentially contaminated through the oral cavity, while one was closed. All patients achieved bone healing (mean, 70.8 days; range, 63–96 days). All patients recovered their pre-traumatic dental occlusion and showed no dental damage as a consequence of the intervention. The only complication observed in all cases was irritation of the oral mucosa at the contact points with the fixator. This resolved favorably within one week without antibiotic treatment. The potential advantages and limitations of the technique compared with existing fixation methods are also discussed.

## Simple Summary

Mandibular fractures in dogs are difficult to treat because they often involve the tooth roots, are frequently open to the oral cavity, and require the accurate restoration of normal dental occlusion. Although several fixation techniques are available, each has its own indications and limitations, and some clinical situations remain challenging to manage. This study describes a novel intraoral fixation technique based on a lingual arch, cerclage wire anchorages, and bis-acrylic resin. The technique was applied in six dogs with eleven mandibular body fractures of different configurations. Bone healing was achieved in all cases, normal dental occlusion was restored and maintained, and no permanent damage to the teeth or mandibular neurovascular structures was observed. The different anchorage configurations allowed for the adaptation of the fixation system to a variety of fracture patterns and dental conditions, including cases with advanced periodontal disease. These preliminary results suggest that this technique may represent a useful alternative for selected mandibular fractures. Further clinical and biomechanical studies are warranted to evaluate its broader clinical application.

## 1. Introduction

The mandibular fracture incidence in dogs reported in the literature varies greatly, accounting for 1.6 to 6% of all canine fractures [1,2]. Animals under one year of age are overrepresented, and males appear to be more affected than females. The most common causes of fracture also differ across authors, with dog fights and road traffic accidents reported as the primary causes. The mandibular body is the most common fracture location, followed by the symphysis and the mandibular ramus, and finally the condyle area. In mandibular body fractures, the molar region (*pars molaris*) is most frequently affected. The first molar and the canine are the most frequently affected teeth. Most reported mandibular fractures are open, implying a risk of the fracture site becoming contaminated. In addition, between 20% and 27% of dogs present multiple mandibular fractures, most commonly involving the mandibular body and symphysis, or occurring as bilateral body fractures [2,3,4].

The mandible has several unique anatomical characteristics that complicate the treatment of fractures. The mandibular body contains the roots of teeth, and these roots occupy more than two-thirds of the dorsal body in the molar region. The ventral third includes the mandibular canal, which contains the innervation and vascular supply of the teeth; ventral to the canal there is only a layer of dense cortical bone [5]. In the incisor region (*pars incisiva*), the roots of the incisor and canine teeth occupy almost the whole bone and are surrounded by a thin layer of bone, which may make securing implants in this area difficult [6].

The principles of treating maxillofacial fractures include maintaining or restoring dental occlusion, providing stable fracture fixation, avoiding iatrogenic damage to the teeth, and promoting the early recovery of oral function [7,8]. Likewise, functional fixation and minimally invasive techniques are recommended whenever possible because they are gentler on the soft tissue and its vascular supply (which is essential to achieve proper bone healing) and reduce the risk of infection [9,10,11]. Treatment for mandibular fracture repair can be characterized as invasive, involving external or internal fixation devices (such as plates, external fixators, pins, and interfragmentary wires) [5,12,13], or minimally invasive, relying on closed reduction and tooth-borne devices (such as acrylic/composite interdental splints, interdental wiring/splints, and maxillomandibular rigid fixation) [14,15,16]. The reported complication rate in the treatment of mandibular fractures is very high [3,4]. Umphlet et al. (1990) reported a 34% complication rate in 157 dogs presenting mandibular fractures, with dental malocclusion (12.1%), osteomyelitis (8,91%), and nonunion (5.73%) being the most common complications [3].

Kern et al. (1993) evaluated the resistance of five minimally invasive intraoral fixation methods ex vivo (Erich arch, Stout loop interdental cerclages, acrylic, acrylic-reinforced Stout Loop, and acrylic-reinforced Erich arch) in mandibles obtained from cadavers with osteotomies between the third and fourth premolars subjected to flexural forces [17]. They determined that the configuration combining the Erich arch with acrylic presented the best mechanical results in terms of flexural strength and stiffness. Subsequently, Kern et al. (1995) evaluated the effectiveness of the same system in artificially created fractures in live animals and compared it with the efficiency of fixation with plates and external fixators in terms of the distance between fragments, alignment, dental occlusion, and healing times, as well as complications in terms of implant loosening and damage to teeth [18]. They concluded that the intraoral fixation described above could be a viable method for repairing mandibular fractures.

The large number of patients affected by mandibular fractures treated in clinical practice; the high complication rate in their treatment; and their repercussions for function, quality of life, and esthetics in the event of treatment failure justify the research and development of new osteosynthesis systems and techniques that are more economical, easier to use, and able to produce satisfactory outcomes with lower complication rates. The purpose of this study was to explore the application of an intraoral fixation system based on the same components reported by Kern et al. (a fixation bar, cerclage wire anchorage, and acrylic reinforcement) for the treatment of 11 mandibular body fractures in six canine patients. The general surgical technique, the results obtained, and the advantages and limitations of this fixation system compared with other treatment options are also discussed.

However, the technique described in the present study incorporates additional anchorage methods, including mandibular bone tunnels and circummandibular cerclage wires. Furthermore, the fixation bar consists of a lingual arch that, in all cases, bridges both mandibles.

## 2. Case Series

This prospective case series included six dogs with fractures of the mandibular body treated between April 2024 and March 2026. All dogs were presented to the Traumatology and Orthopaedics Service of the Veterinary Teaching Hospital of the University of Zaragoza.

The inclusion criteria were a fracture of the mandibular body located rostral to the first molar or involving the alveolus of the first molar, the presence of permanent dentition, and clinical follow-up until fracture healing was confirmed and the intraoral fixator was removed, including a final clinical examination two weeks after fixator removal.

All patients that presented during the study period and met the inclusion criteria were included in the case series and were treated using the intraoral fixation technique described in this manuscript. No patient was excluded based on the condition of the teeth or mandibular bone at the time of presentation. No patient was excluded from the case series due to a lack of follow-up until removal of the intraoral fixator or failure of the technique.

Three surgeons performed the surgical procedures1: One surgeon is a European Board of Veterinary Specialisation (EBVS) specialist and a European Veterinary Dental College (EVDC) Diplomate in Veterinary Dentistry. The two surgeons are accredited in Traumatology and Orthopaedics by the Spanish Small Animal Veterinary Association (AVEPA).

### All Cases

After presentation, a clinical examination and radiological study of the oral cavity, including laterolateral and ventrodorsal projections, were performed under sedation or anesthesia. Computed tomography (CT) studies were performed in Cases 1 and 2 using a conventional fan-beam CT scanner (Revolution CT; GE HealthCare, Milwaukee, WI, USA) to more comprehensively assess these cases and to accurately illustrate the surgical technique described in this study. The pre-existing type of occlusion before the fractures was determined by manual fracture reduction. The fractures were classified (Table 1) according to their complexity (simple, complex, or comminuted), their location within the mandibular body (molar or incisive region), their communication with the oral cavity or the exterior (closed or open), and, according to Kahnberg and Ridel (1979), the relationship between the fracture line and the tooth root and its apex [19]. The conditions of the periodontium and the teeth were recorded to compare them with those after the removal of the intraoral fixator.

All patients were premedicated with methadone (Semfortan^®^, Eurovet Animal Health B.V., Bladel, The Netherlands, 0.2–0.3 mg/kg IM) and dexmedetomidine (Dexdomitor^®^, Orion Corporation, Espoo, Finland, 2–5 µg/kg IM); anesthesia was induced with propofol (Propovet^®^, Zoetis Spain, Madrid, Spain, 4–6 mg/kg IV to effect) and maintained with isoflurane (IsoFlo^®^, Zoetis, Madrid, Spain, 1.2–2%) in oxygen. Antibiotic prophylaxis was administered 45 min prior to surgery using intravenous cefazolin (Cefazolina Normon^®^, Laboratorios Normon, S.A., Tres Cantos, Madrid, Spain, 22 mg/kg IV).

Endotracheal intubation was performed via pharyngostomy. This kind of intubation allowed the tongue to be displaced caudally toward the oropharynx to improve visualization of the mandibular arch, facilitate fracture reduction and stabilization, and control occlusion intraoperatively.

After intubation, patients were placed in the sternal recumbency position. Intraoral antisepsis was performed immediately before surgery using diluted chlorhexidine solution (Peridex^®^, 3M ESPE, St. Paul, USA, 0.12%) applied to the oral cavity. To allow mouth opening and closure during surgery, a gauze roll strip was placed caudally to the maxillary canines and tied to a bar over the head, leaving the head suspended with both mandibles free. Manual traction of the jaw was performed instead of using mouth openers because the authors believe that mouth openers exert pressure and an uncontrolled distraction that can increase damage to soft tissues or the fracture site.

**Figure 1:**
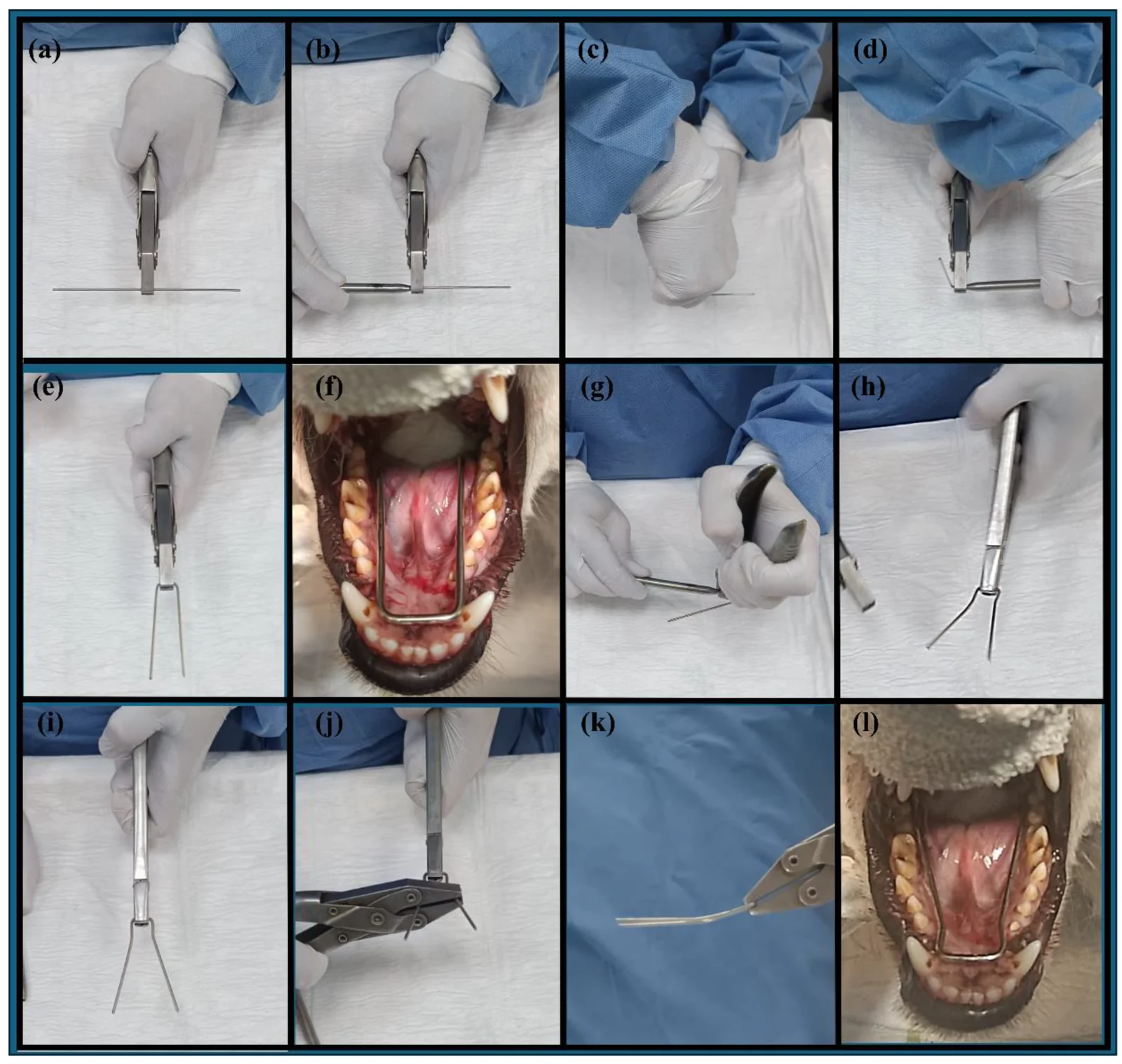
Step-by-step preparation of the lingual arch used as the connection bar of the intraoral fixation system described in this study. A Kirschner wire of appropriate diameter, selected according to the patient’s size, is contoured using pliers and a K-wire bender. The first step consists of creating a U-shaped configuration (**a**–**e**). To achieve this, the Kirschner wire is grasped at its midpoint and bent approximately 90° on each side using the K-wire bender. The central portion of the U is contoured to adapt to the lingual surface of the mandibular incisor region, while both arms are contoured to follow the lingual surfaces of the canine, premolar, and molar regions of each mandible. The U-shaped arch is then checked to ensure that it is of sufficient length to extend at least to the level of the first molar (**f**). Subsequently, both arms of the U are progressively flared laterally to adapt to the lingual surfaces of the premolar and molar regions of each mandibular bone (**g**–**i**). The arms are then contoured to reproduce the natural lateral curvature of the mandible when viewed laterally (**j**,**k**). Finally, the completed lingual arch is checked to ensure accurate adaptation along the lingual aspect of the alveolar margin before its clinical application (**l**).

Subsequently, an arch-shaped connecting bar (lingual arch) (Figure 1) was made with Kirschner wire of variable diameter depending on the size of the patient (1.2–2 mm in this case series). Using preoperative X-rays as a reference and by direct visualization of the oral cavity, this arch was precontoured intraoperatively to adapt it to the lingual aspect of the mandibles and teeth as close as possible to the alveolar margin, paying special attention to those areas where it would be anchored to the teeth or bone. The arch-connecting bar was extended bilaterally along both mandibles at least to the caudal aspect of the first molars.

The connecting bar was anchored to the jaw using simple loop cerclage wires (0.6–0.8 mm in this case series), which were placed in three different ways.

**Figure 2:**
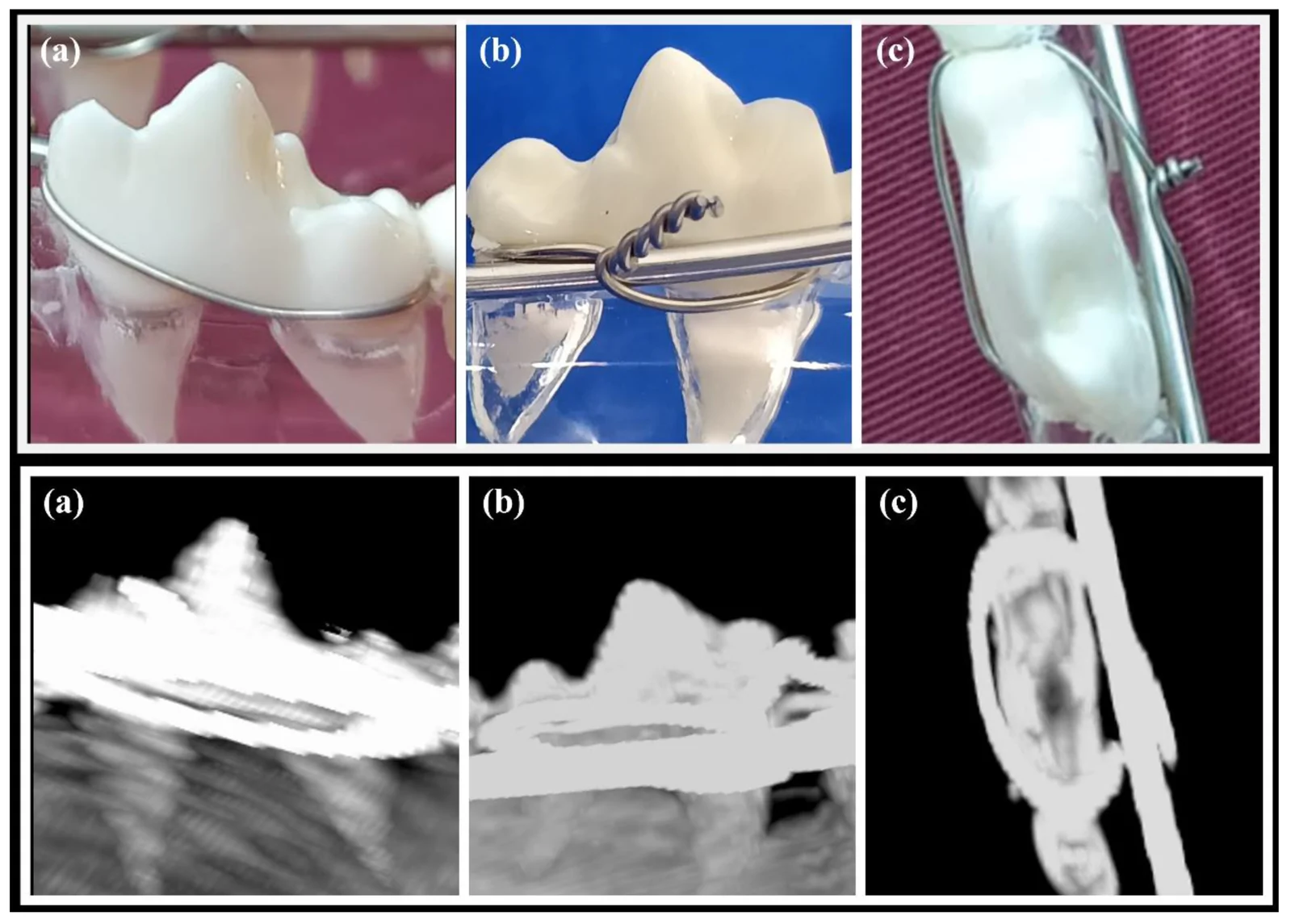
Buccal (**a**), lingual (**b**), and dorsal (**c**) views of a Type A anchorage placed on the first molar using a plastic model (upper panel). The lingual arch is adapted to the lingual surface of the tooth and positioned adjacent to the mandibular alveolar border after tensioning of the cerclage loop around the cervical portion and crown of the anchor tooth. The same views are reproduced using MIP images obtained from the CT study of Case 1 (lower panel).

**Figure 3:**
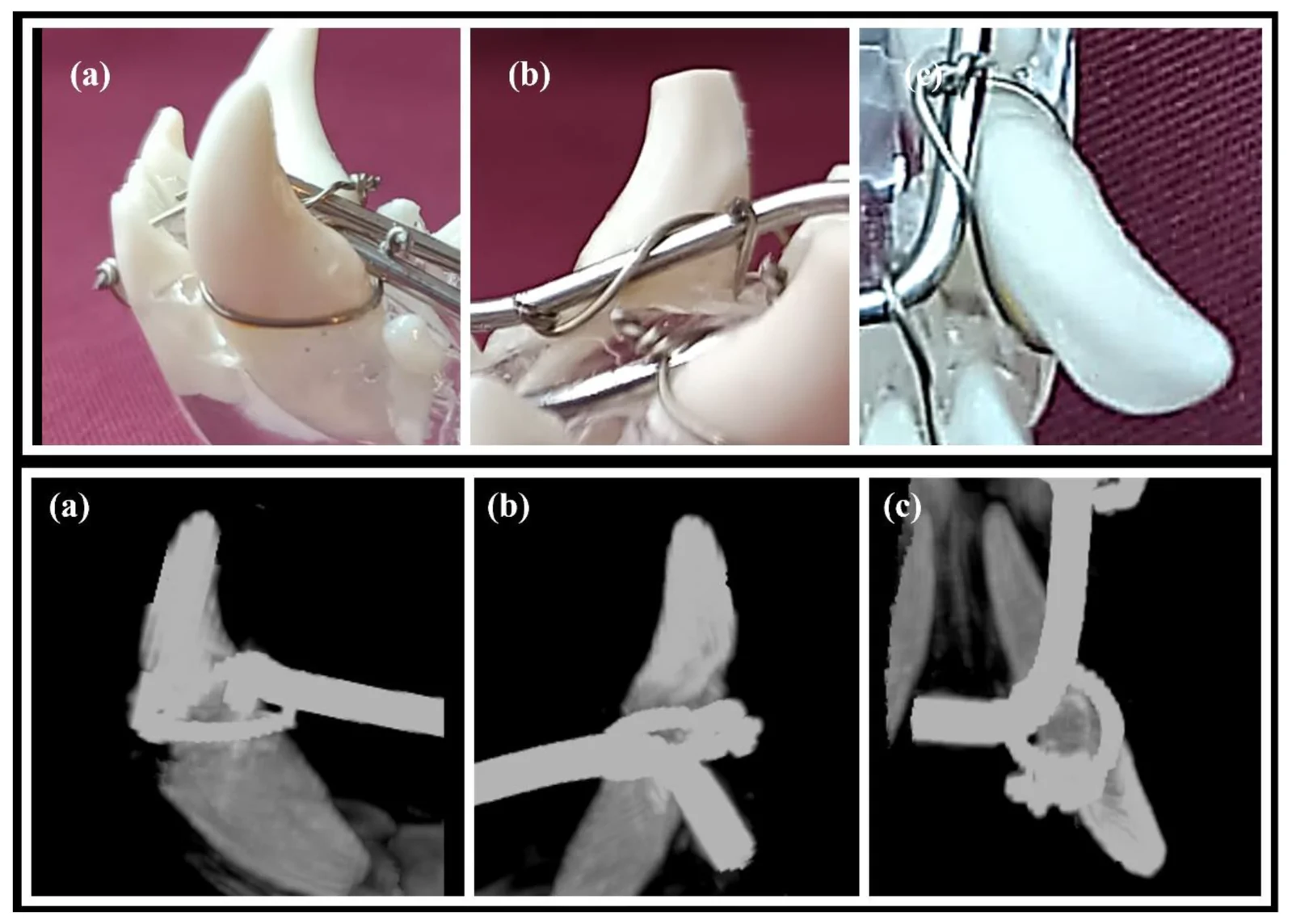
Labial (**a**), lingual (**b**), and dorsal (**c**) views of a Type A anchorage placed on the canine tooth using a plastic model (upper panel). The lingual arch is adapted to the lingual surface of the tooth and positioned adjacent to the mandibular alveolar border after tensioning of the cerclage loop around the cervical portion and crown of the anchor tooth. The same views are reproduced using maximum intensity projection (MIP) images obtained from the computed tomography study of Case 2 (lower panel).

The first way was surrounding the necks and crowns of the teeth (Type A anchor: tooth-bar cerclage wire) (Figure 2 and Figure 3). In all cases where healthy teeth were present, and the crowns and neck size and shape allowed cerclage wire placement, Type A anchorage to the teeth was chosen. Canines, fourth premolars, and first and second molars are preferred for this kind of anchorage because of their size and shape. When premolars or molars were anchored, the passage of the cerclage was facilitated by a hypodermic needle (20–23 G) that was passed through the mucosa adjacent to the neck of the tooth. After passing the cerclage wire around the cervical portion of the anchor tooth and the lingual arch, both free ends of the wire are grasped with wire-twisting pliers and rotated together. As the pliers are turned, the loop progressively tightens, drawing the lingual arch closer to the tooth until it is firmly adapted against its lingual surface. Once the cerclage has been adequately tightened and twisted, the excess wire ends are cut and the remaining twisted segment is bent and closely adapted to the tooth surface. Whenever possible, the cerclage twists are positioned on the lingual aspect of the tooth. However, in small patients, access to the lingual surface is often limited; therefore, the twists are preferentially placed on the labial or buccal aspect of the tooth to facilitate the procedure.

**Figure 4:**
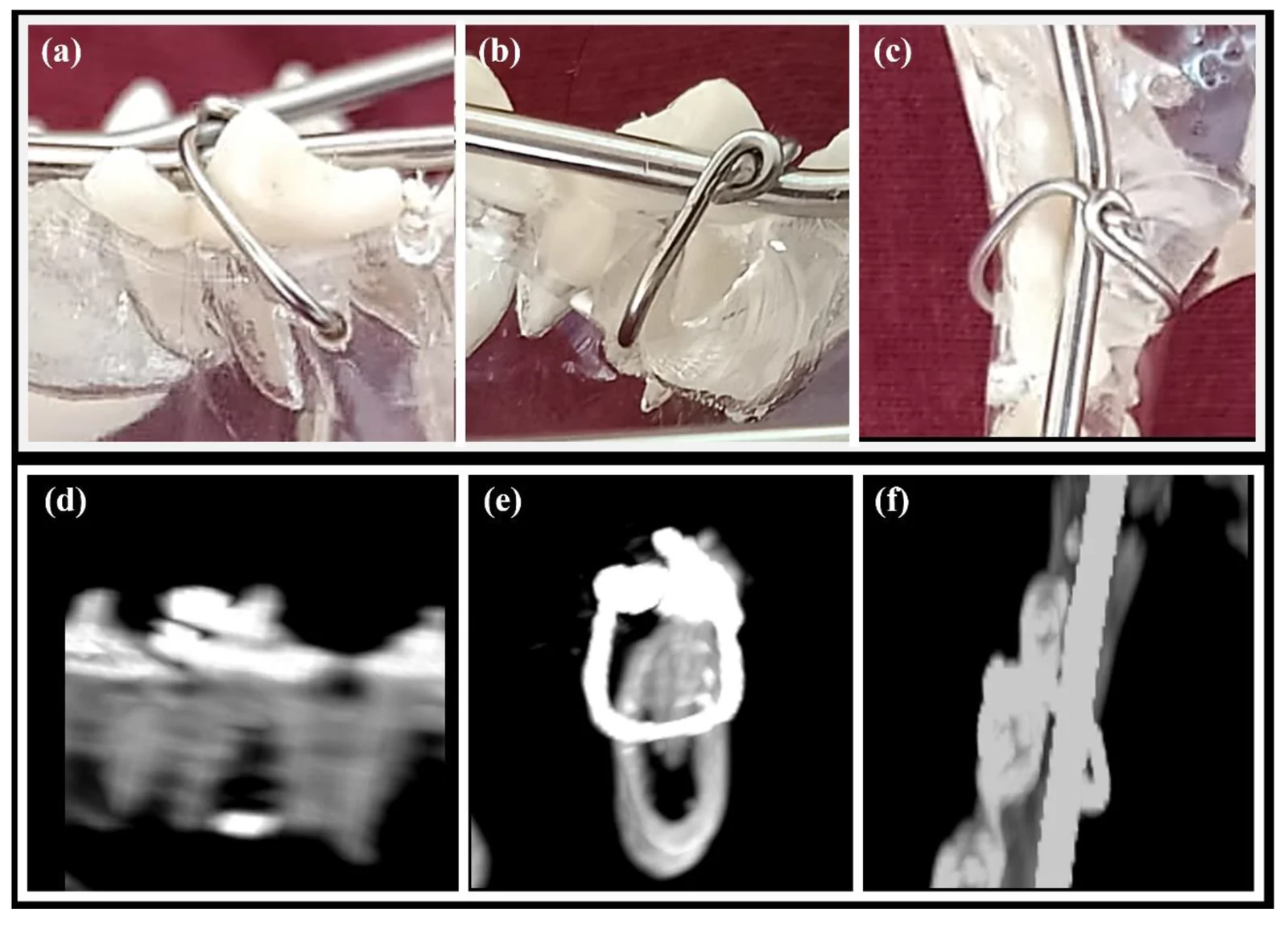
Labial (**a**), lingual (**b**), and dorsal (**c**) views of a Type Bf anchorage placed at the second premolar on a plastic model (upper panel). The anchorage is created by passing a cerclage wire through a small osseous tunnel located between the roots of the tooth. The wire is then passed around the lingual arch to form a loop, which is tightened and twisted, with the cerclage twist preferentially positioned on the lingual aspect. Lingual (**d**), frontal (**e**), and dorsal (**f**) views of the same Type Bf anchorage reproduced from maximum intensity projection (MIP) reconstructions of the CT study of Case 1 (lower panel). The course of the cerclage wire through the mandibular bone can be observed, demonstrating that its trajectory preserves both the adjacent dental roots (**d**) and the neurovascular structures of the mandibular canal (**e**).

The second way was through small mandibular bone tunnels (Type B anchor: mandibular bone hole-bar cerclage wire) crossing mandibular bone between the roots of a tooth (Type Bf) (Figure 4) or between the roots of two adjacent teeth (Type Bi). Type B anchorage was employed when the cerclage wire could not be anchored to the tooth because it did not adapt well to the tooth neck or crown, slid, or did not feel stable enough. This anchorage configuration was most commonly used in the premolar region and the incisor region of small dogs, in which the morphology and size of the incisors and the first, second, and third premolars were unsuitable for the placement of a Type A anchorage. The bone tunnels for Type B anchors were made with a small-diameter drill bit (0.8–1.1 mm) using an orthopedic drill and a drilling-guide, drilling mandibular bone between the roots of the same tooth or between adjacent teeth roots, and always respecting the neurovascular structures of the mandibular canal and the tooth roots. The bone tunnel was drilled from the labial to the lingual aspect of the mandible. To accurately position the tunnel, measurements obtained from digital radiographs or, when available, computed tomography images, were used to position the guide and accurately perform the drilling, minimizing the risk of damage to the dental roots or invasion of the mandibular canal. Current digital radiography allows precise measurements to be obtained for planning the positions of these bone tunnels. In addition, previously published surgical techniques involving similar bone perforations were used as a guide to perform the bone tunneling required for Type B cerclage anchorage. These included techniques for treating mandibular fractures using plates and miniplates [20], as well as intermandibular cerclage and interosseous cerclage techniques [21]. The correct positioning of the bone tunnels was confirmed by radiography and, when available, computed tomography (CT).

After passing the cerclage wire through the previously created osseous tunnel and around the lingual arch, the loop is completed by passing the wire dorsally to the mandibular alveolar border, through the interdental space of the teeth. As with Type A anchorages, both free ends of the wire are grasped with wire-twisting pliers and twisted together to progressively tighten the loop, securing the lingual arch firmly. Once adequate tension has been achieved, the excess wire ends are cut and the remaining twisted segment is bent and adapted closely to the tooth surface. Whenever possible, the cerclage twist is positioned on the lingual aspect; however, in small patients, where access to the lingual surface is limited, the twist is preferentially placed on the labial aspect.

**Figure 5:**
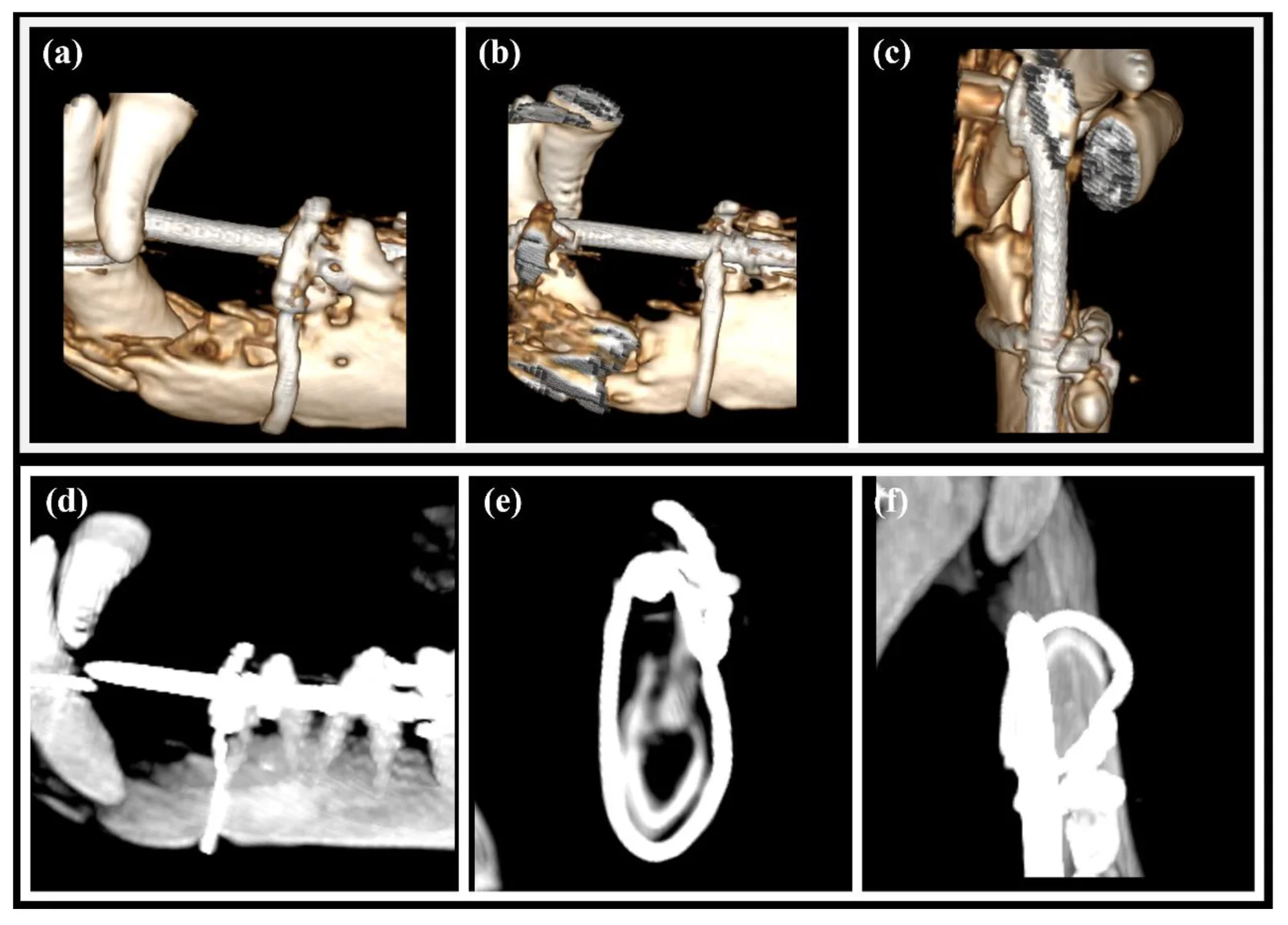
Three-dimensional reconstructions of the CT study of Case 2 showing a Type C anchorage placed in an edentulous premolar region. Labial (**a**), intraoral (**b**), and dorsal (**c**) views are shown in the upper panel. The cerclage wire is passed around the body of the mandible, exiting through the labial and lingual aspects. The loop is completed by passing the wire around the lingual arch, after which the cerclage is tightened and twisted to secure the fixation. Maximum intensity projection (MIP) reconstructions of the same Type C anchorage are shown in the lower panel, including labial (**d**), frontal (**e**), and dorsal (**f**) views.

The third way was surrounding the mandibular bone (Type C anchor: circummandibular-bar cerclage wire) (Figure 5). The Type C anchor was used in areas with mobile teeth, missing teeth, or evident radiological mandibular bone loss due to advanced periodontal disease. In these cases, the cerclage wire is passed around the jaw, again using a hypodermic needle as a guide, and leaving the knot in an intraoral position close to the connecting bar.

At least two rostral and two caudal cerclages remained at each fracture side in every case. Once all the anchors were in place and surrounding the connecting bar, each one was gradually tightened, controlling the fracture reduction and mouth occlusion throughout the process. Once the tightening of the cerclage wires was complete, the occlusion was checked.

A bis-acrylic resin (Protemp™ 4, 3M ESPE AG, Seefeld, Germany) was applied to cover the connecting bar, cerclage wires, and teeth in the connecting areas. The extent of coverage depended on the surgeon’s preference, but all anchorage areas including the connecting bar, cerclage wires, and necks of the teeth involved were always completely covered. An effort was made to avoid the excessive accumulation of resin on the occlusal surface of the teeth and ensure proper occlusion during resin placement; excess resin was removed using a burr.

The postoperative occlusion was checked and recorded photographically, and a postoperative radiographic study including laterolateral and ventrodorsal views of the head was also performed.

Postoperative medication during the hospitalization period consisted of Methadone 0.3 mg/kg IV q6h (Semfortan^®^, Eurovet Animal Health B.V., Bladel, The Netherlands), cefazolin 22 mg/kg IV q12h (Cefazolina Normon^®^, Laboratorios Normon, S.A., Tres Cantos, Madrid, Spain), and meloxicam 0.2 mg/kg SC q24h (Metacam^®^, Boehringer Ingelheim Vetmedica GmbH, Ingelheim am Rhein, Germany) and IV maintenance fluid therapy (Lactated Ringer’s solution, B. Braun Melsungen AG, Melsungen, Germany). No cases required an esophageal feeding tube, and all the patients were discharged within 24 h after surgery, being able to drink and eat normally.

Postoperative medication after discharge was administered for 5 days and consisted of oral cephalexin 22 mg/kg PO q12h (Cefabactin^®^, Le Vet Beheer B.V., Oudewater, The Netherlands) and meloxicam 0.1 mg/kg PO q24h (Meloxoral^®^, Le Vet Beheer B.V., Oudewater, The Netherlands. Postoperative recommendations included a soft diet, water ad libitum, and the strict avoidance of ball games or chewing toys. Owners were instructed to routinely clean the oral cavity by flushing with saline solution throughout the period in which the intraoral fixation device remained in place.

All patients underwent radiographic follow-up examinations every four weeks after surgery until bone healing was confirmed. Bone healing was considered to have occurred when fracture union was radiographically demonstrated either by callus formation or by direct cortical bridging between the cortices adjacent to the fracture site. In Cases 1 and 2, bone healing was additionally confirmed by computed tomography (CT).

**Figure 6:**
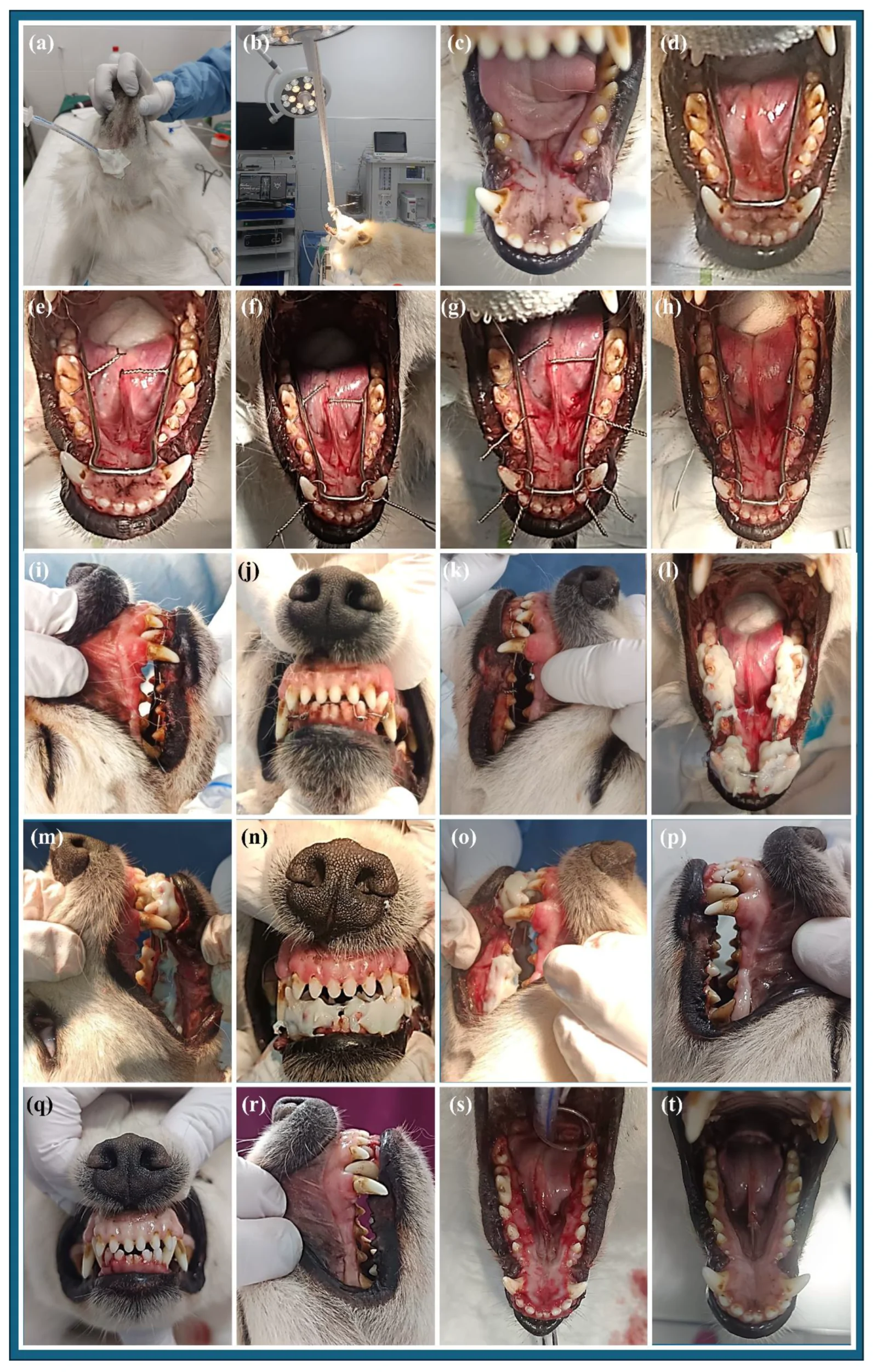
Photographic record of Case 1. Endotracheal intubation was performed using a transpharyngeal approach (**a**). The patient was positioned in sternal recumbency with the head suspended using a bandage (**b**). Bilateral mandibular body fractures open towards the oral cavity were present, with displacement of the rostral mandibular dental arcade and (**c**). (**d**–**h**) Fixation of the lingual arch to the mandible. The lingual arch was contoured to match the morphology of the lingual aspect of the mandible, where it would be positioned (**d**). Type A anchorages were placed around both first molars (**e**) and both canine teeth (**f**). Additional fixation was achieved using Type A anchorages around both third incisors and Type B-F anchorages placed between the roots of both third premolars (**g**). After all anchorages were placed, the wire ends were bent to avoid interference with the tongue or lips (**h**). Restoration of normal occlusion following fixation with the lingual arch and cerclage wires is shown in the right lateral (**i**), frontal (**j**), and left lateral (**k**) views. Bis-acrylic resin was applied to cover the lingual arch and the anchored teeth (**l**). Normal occlusion was then reconfirmed in the right lateral (**m**), frontal (**n**), and left lateral (**o**) views. After the radiographic confirmation of fracture union, the intraoral fixation was removed. Normal occlusion was again confirmed in the left lateral (**p**), frontal (**q**), and right lateral (**r**) views. At the time of fixation removal, mild irritation of the oral mucosa was present at the contact points with the appliance (**s**). This was treated by the local application of chlorhexidine and had completely resolved one week later (**t**).

**Figure 7:**
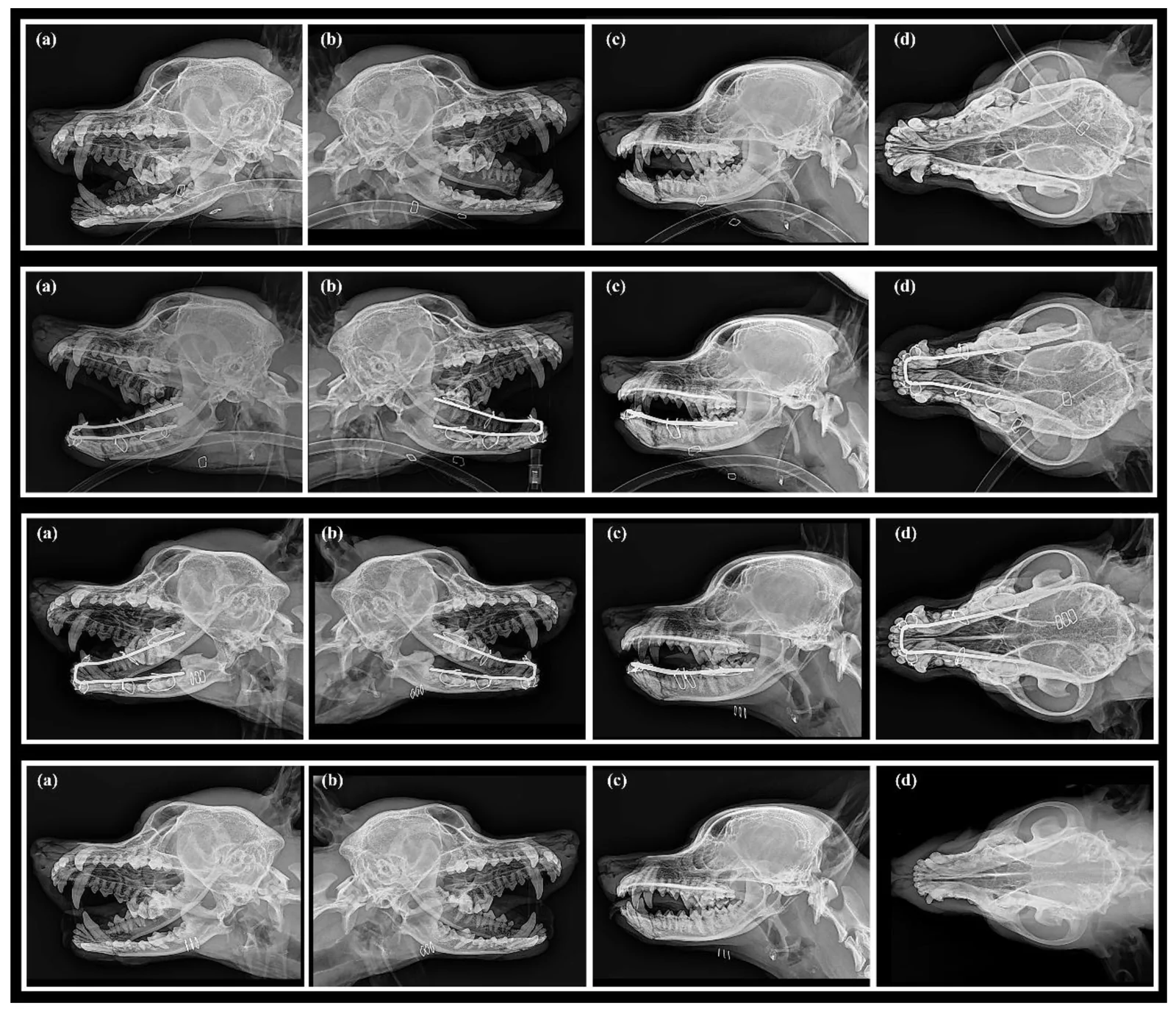
Radiographic evaluation of Case 1. In each panel, the radiographic projections are arranged as follows: (**a**) right oblique lateral projection, (**b**) left oblique lateral projection, (**c**) right laterolateral projection, and (**d**) dorsoventral projection. The upper panel shows the preoperative radiographic study, demonstrating fractures affecting both mandibular bones and resulting in rostroventral displacement of the incisive portion of the mandible. The upper middle panel shows the immediate postoperative radiographic study following the reconstruction and fixation of both fractures using the lingual arch, cerclage wires, and bis-acrylic resin. The lower middle panel shows the radiographic follow-up at 8 weeks, showing bone healing of both fractures. The lower panel shows the radiographic evaluation obtained immediately after removal of the fixation construct.

**Figure 8:**
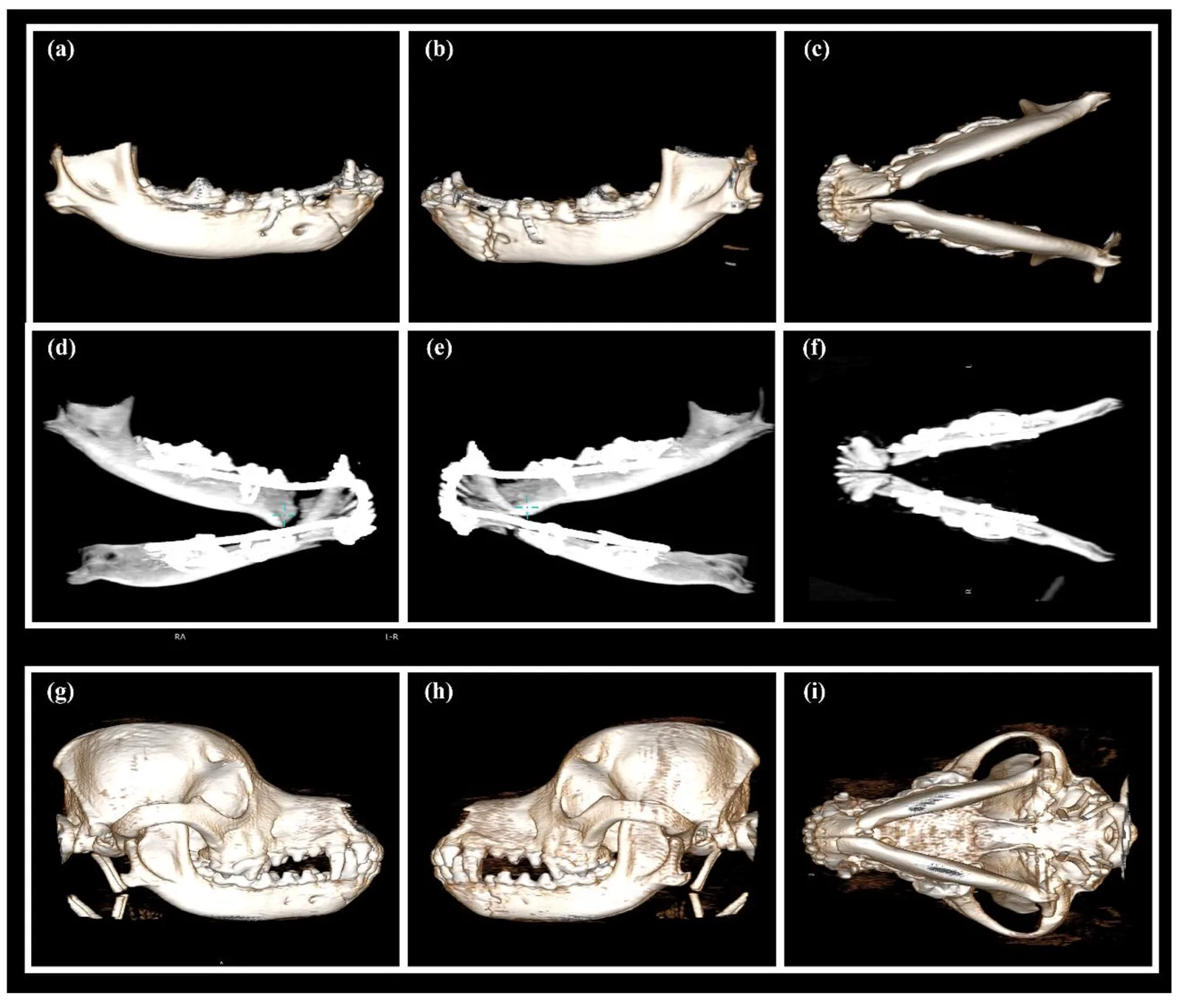
Computed tomography evaluation of Case 1. The upper panel shows computed tomography images of the mandibular body after segmentation from the remainder of the skull, including three-dimensional reconstructions in the right lateral (**a**), left lateral (**b**), and ventral (**c**) views, and MIP reconstructions in a left oblique lateral (**d**), right oblique lateral (**e**), and dorsal (**f**) view. These images illustrate the overall configuration of the fixation construct, including the lingual arch, the dental and mandibular anchorage points, and fracture reduction. The lower panel shows three-dimensional reconstructions of the skull obtained after removal of the fixation construct—(**g**) right lateral view, (**h**) left lateral view, and (**i**) ventral view of the skull—demonstrating the restoration of normal occlusion.

Case 1 (Figure 6, Figure 7 and Figure 8)—an adult, intact male, 6.2 kg Pomeranian dog—was presented with a 72 h history of pain, bleeding, and difficulty closing the mouth following a fight with another dog. Clinical, radiographic, and computed tomographic examination of the oral cavity revealed two simple mandibular body fractures, both open towards the oral cavity. The left mandibular fracture was an alveolodental fracture between the canine and the first premolar (alveolodental type VI). The right mandibular fracture was located in the rostral portion of the mandible and involved the canine (alveolodental type I). As a consequence of the fractures, the rostral portion of the mandibular dental arcade was severely displaced, resulting in malocclusion. The teeth were intact, and periodontal disease was present (stage 2).

Fixation was performed using a 1.5 mm diameter lingual arch extending bilaterally caudal to both second molars and secured with a 0.8 mm diameter cerclage wire. Type A anchorages were placed around both first molars, both canines, and both third incisors. Type B anchorages were positioned through osseous tunnels located between the roots of both third premolars. Bis-acrylic resin was applied to cover the lingual arch, the cerclage wires, and the connection areas extending from both first molars to both third premolars and from both first premolars to the first incisor. After fixation, normal occlusion was confirmed to have been restored. Bone union was achieved, with cortical bridging, 64 days after fixation.

**Figure 9:**
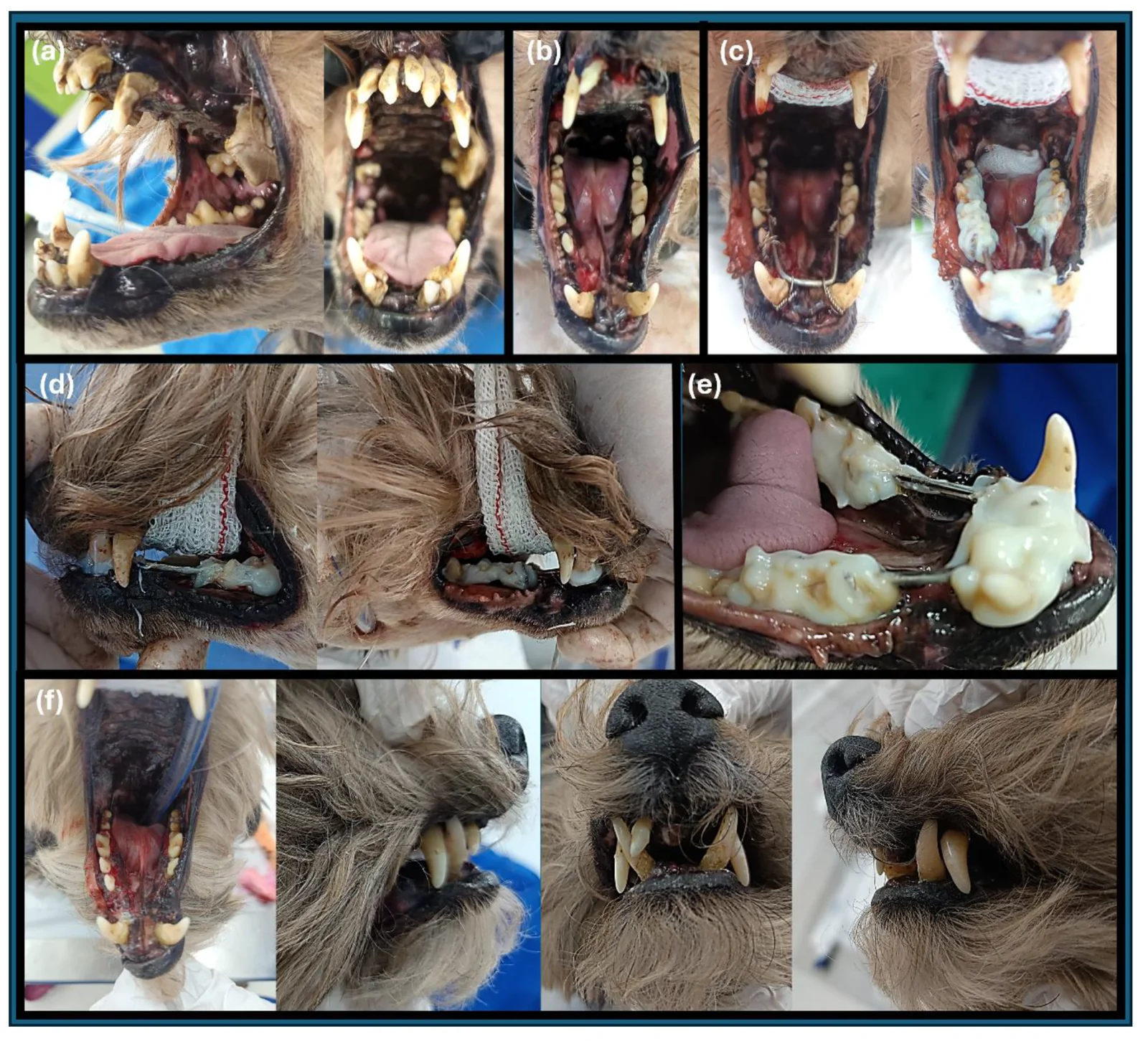
Photographic record of Case 2. The patient presented with severe periodontal disease and a rostral fracture of the right mandible (**a**). Oral prophylaxis, consisting of ultrasonic scaling and extraction of mobile teeth, was performed (**b**). An intraoral lingual arch was applied and anchored to the teeth using cerclage wire, and the fixation was reinforced with bis-acrylic resin (**c**) (from left to right, respectively). Left and right lateral views showing restoration of normal dental occlusion immediately after surgery (**d**). Fourteen days after surgery, the oral mucosa had covered the wound at the fracture site (**e**). Following removal of the intraoral fixation, no damage to the teeth was observed and normal dental occlusion was maintained (**f**).

**Figure 10:**
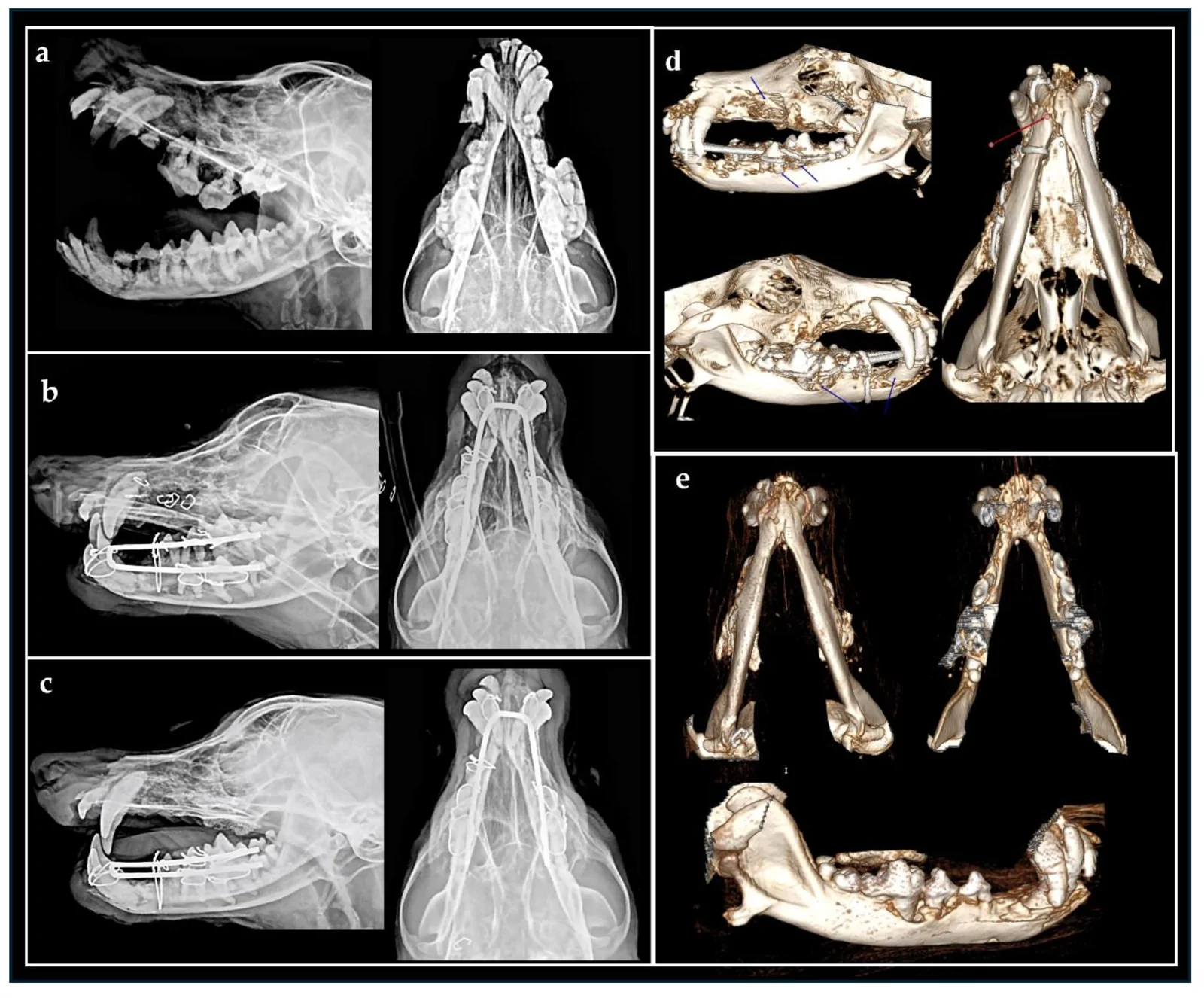
Radiographic and CT study of Case 2. Preoperative (**a**), postoperative (**b**), and 12-week follow-up (**c**); orthogonal right lateral and dorsoventral radiographic projections are shown, with bone healing. The postoperative CT study revealed alveolar bone loss, particularly affecting the canine teeth, fourth premolars, and first molars ((**d**), left and right lateral views, blue arrows); fracture reduction after application of the intraoral fixation ((**d**), ventral view, red arrow); and the overall configuration of the intraoral fixation and the restored normal occlusion ((**d**), all images). Following removal of the intraoral fixation, CT confirmed bone healing, preservation of the remaining teeth, and maintenance of normal occlusion ((**e**), ventral, intraoral, and right lateral views).

Case 2 (Figure 9 and Figure 10)—an 8-year-old, 6 kg, male Yorkshire Terrier crossbreed dog—was presented with a 48 h history of sudden onset of oral pain, mild bleeding, and difficulty closing the mouth after playing with a ball. Clinical, radiographic, and computed tomographic examination of the oral cavity revealed malocclusion caused by an open mandibular fracture, open towards the oral cavity, located in the rostral region of the right mandible and involving the canine tooth (alveolodental type I). The fracture was associated with loss of mandibular and maxillary bone adjacent to the dental alveoli, predominantly affecting the canine, fourth premolar, and first mandibular molar teeth. Stage IV periodontal disease was present and led to the fracture being classified as pathological. Teeth 103, 105, 106, 107, 108, 205, 206, 207, 210, 301, 401, 402, and 405 were absent. Several of the remaining teeth were mobile.

Periodontal treatment was performed by extracting the mobile teeth affected by stage IV periodontal disease. The remaining teeth (102, 104, 204, 304, 308, 309, 311, 404, 408, 409, 410, and 411) were preserved and treated by ultrasonic scaling and dental polishing.

Fixation was performed using a 1.5 mm diameter intraoral lingual arch extending bilaterally caudal to the first molars and anchored to the mandible with a 0.8 mm diameter cerclage wire using different anchorage configurations. Type A anchorages were placed around both first molars, both fourth premolars, and both canine teeth. A Type Bf anchorage was placed through an osseous tunnel between the roots of the right third premolar. A Type C anchorage was placed rostral to the right third premolar. Bis-acrylic resin was applied to cover the lingual arch, the cerclage wires, and the connection areas extending from the left second molar to the left fourth premolar, from the left canine to the right canine, and from the right third premolar to the right second molar. After fixation, normal occlusion was confirmed to have been restored. Bone union was achieved, with cortical bridging, 96 days after treatment.

**Figure 11:**
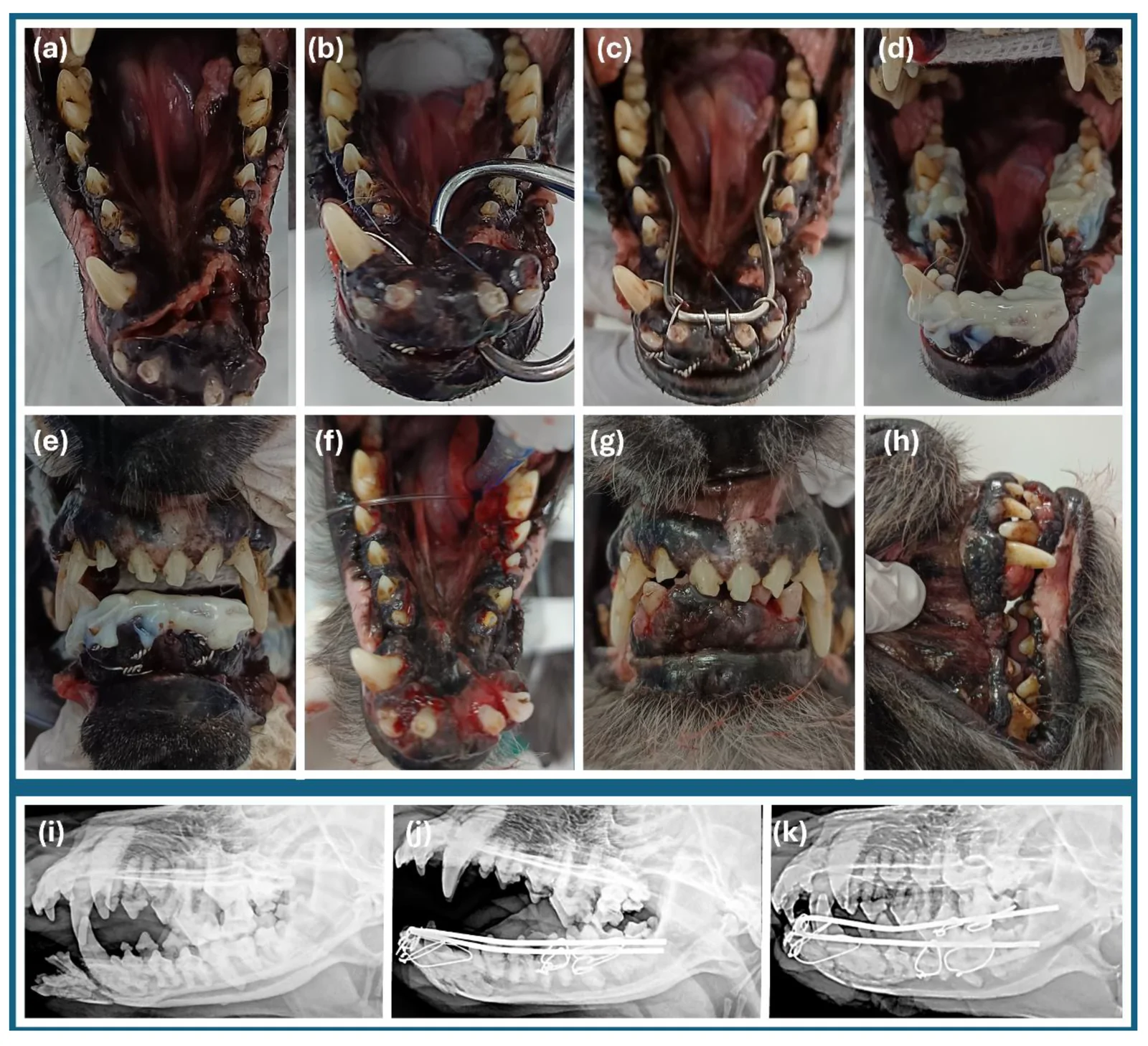
Photographic record (upper panel) and radiological study (lower panel) of Case 3. An open fracture of the rostral portion (**a**,**i**) was treated with two cerclages after reduction with a two-point reduction forceps (**b**) and with a lingual arch anchored to the mandible using cerclages to the tooth crowns and through bone tunnels between the tooth roots (**c**); the anchorage was reinforced using bis-acrylic resin (**d**,**j**). Stable fixation and restoration of occlusion were achieved (**e**), which led to bone healing at 67 days (**k**). After fixator removal, occlusion continued to be normal (**g**,**h**) and no changes were found in the mouth except for mild irritation of the gingiva that touched the device (**f**).

Case 3 (Figure 11)—a 14-year-old, neutered male, 14 kg mixed-breed dog—was presented 48 h after onset of pain, bleeding, and difficulty closing the mouth after a fight with another dog. The patient presented with a simple fracture opened towards the oral cavity that extended through the incisive region of both mandibular bones, affecting the alveoli of the left canine, which was luxated due to the trauma (alveolodental fracture type I) and right canine (alveolodental fracture type II). The rostral mandible bone segment was mildly displaced rostrally, which caused malocclusion. Left and right first incisors were absent, although these appeared to be absent prior to the trauma. The crowns of the incisors were worn, and the patient presented with periodontal disease (stage II).

Reduction was performed using a two-point reduction forceps. Fixation was performed using two simple loop cerclage wires of 0.6 mm. The first cerclage wire encircled the incisive region of the right mandibular bone, passing distally to the neck of right canine, adjacent to the labial and lingual gingiva, and through a bone tunnel located medial to the root of right second incisor. The second cerclage wire encircled the incisive region of the left mandibular bone, passing caudal to the mandibular symphysis, adjacent to the labial surface of the mandibular bone and to the lingual gingiva, and interproximally between the left second and third incisors. Additional fixation was performed using a 1.5 mm diameter lingual arch that extended just caudal to the first molars. The arch was anchored to the mandibular jaw with 0.6 mm diameter cerclage wire using Type A anchors to the left and right molars, and Type B anchors between the left fourth premolar and first molar, right second and third incisors, and fourth right premolar and first molar, between the root of third left incisive and the alveolus of the left canine, and medial to the root of the second left incisor. Resin was applied covering the bar, the cerclages, and the connection areas with teeth from the left molar to the left fourth premolar, from the third left incisive to the right canine, and from the right molar to the right fourth premolar. After fixation, normocclusion was restored. Bone healing was completed 67 days after surgical treatment.

**Figure 12:**
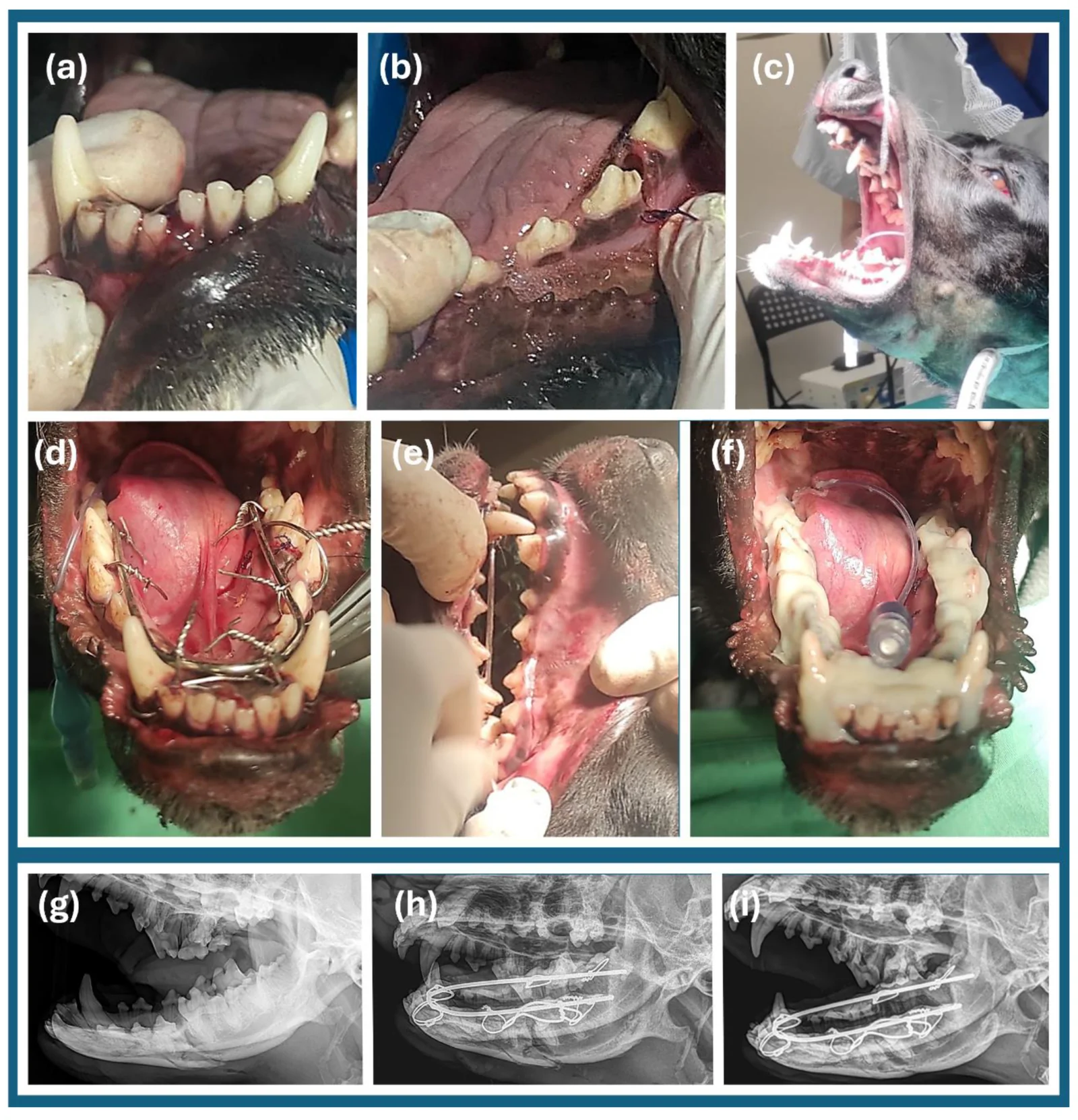
Photographic record (upper panel) and radiological study (lower panel) of Case 4. The patient presented separation of the mandibular symphysis (**a**) and a fracture between teeth 309 and 308 (**b,g**). Routine endotracheal intubation via pharyngostomy and head suspension were performed (**c**), which facilitated fixation using two interdental cerclage wires and a lingual arch anchored to the teeth with Type A anchorage (**d**,**h**). After confirmation of the restoration of normocclusion (**e**), the system was reinforced with bis-acrylic resin (**f**), resulting in bone healing within 71 days (**i**).

Case 4 (Figure 12)—a 4-year-old, intact female, 22 kg mixed-breed dog—was presented 48 h after the onset of pain and difficulty closing the mouth after falling down a ravine. The patient presented with separation of the mandibular symphysis and two fractures in the molar region of the left mandibular bone. The rostral fracture was a closed simple fracture affecting the third premolar (alveolodental type III). The caudal fracture was simple and open toward the oral cavity and affected the fourth premolar (alveolodental type VI). All mandibular teeth were present, and no periodontal disease was detected.

Fixation was performed using two figure-of-eight interdental cerclage wires between the left first molar and left fourth premolar and between the right and left canines using 1.0 mm and 0.8 mm wires, respectively. A 2.0 mm diameter lingual arch was used, which was anchored to the left second molar, left third premolar, left and right canines, right fourth premolar, and right second molar using Type A anchors exclusively, due to the presence of large healthy teeth, with a 0.8 mm diameter wire. The resin covered the bar, the cerclages, and the tooth necks from the second molars of each mandible. After fixation, normocclusion was restored. Bone healing was completed, with callus formation, 71 days after surgical treatment.

**Figure 13:**
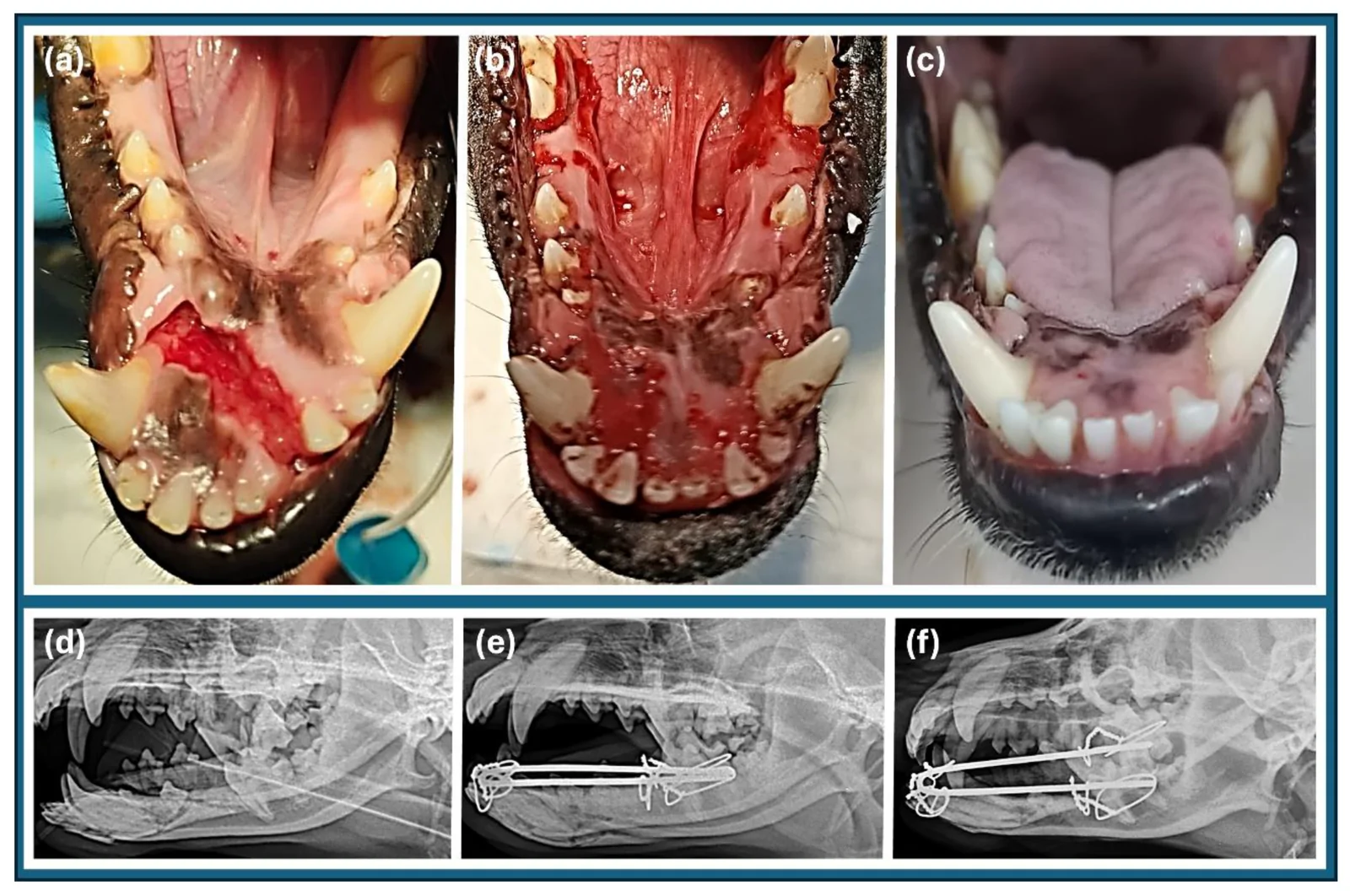
Photographic record (upper panel) and radiological study (lower panel) of Case 5. The patient presented a rostrally displaced bone segment due to fractures between teeth 301 and 302 and at the alveolus of tooth 404 (**a,d**). The fractures were treated using a lingual arch, fixed to the dental necks and to the mandibular bone by a cerclage wire reinforced with bis-acrylic resin (**e**), which resulted in bone healing within 64 days (**f**). After removal of the fixator, the gingiva showed mild irritation (**b**), which resolved within one week (**c**).

Case 5 (Figure 13)—a 2-year-old. intact male, 7 kg Bodeguero Andaluz dog—was presented 48 h after onset of pain, bleeding, and difficulty closing the mouth after an off-leash walk in the countryside; the owners presumed a fight with another dog as the origin of the trauma. The patient presented with two fractures opened towards the oral cavity in the incisive region of the mandible: one simple fracture located between the left first and second incisors and another second complex fracture, with one bone segment, affecting the right canine (alveolodental type I). The first fracture extended across the incisive portion and merged with the second fracture. The mandibular segment rostral to the fracture was displaced rostrally, causing malocclusion. The patient did not have fourth and second left premolars or a fourth right premolar, although their absence did not appear to be related to the trauma; the crowns of the remaining teeth were healthy except for the crown of the right canine, which was fractured. The patient did not present periodontal disease.

Fixation was performed using a 1.5 mm diameter lingual arch that extended bilaterally caudal to the first molars. The anchorage was made with a 0.8 mm diameter cerclage wire using Type A anchors to the first molars and canines, and Type Bi anchors between the left third premolar and first molar, left second and third incisors, right second and third incisors, and right third premolar and first molar. Resin was applied covering the arch, the cerclages, and the connection areas with teeth from the left mandibular first molar to the left third premolar, from the left to the right canine, and from the right first molar to the right third premolar. After fixation, normocclusion was restored. Bone healing was completed, with cortical bridging, 64 days after surgical treatment.

**Figure 14:**
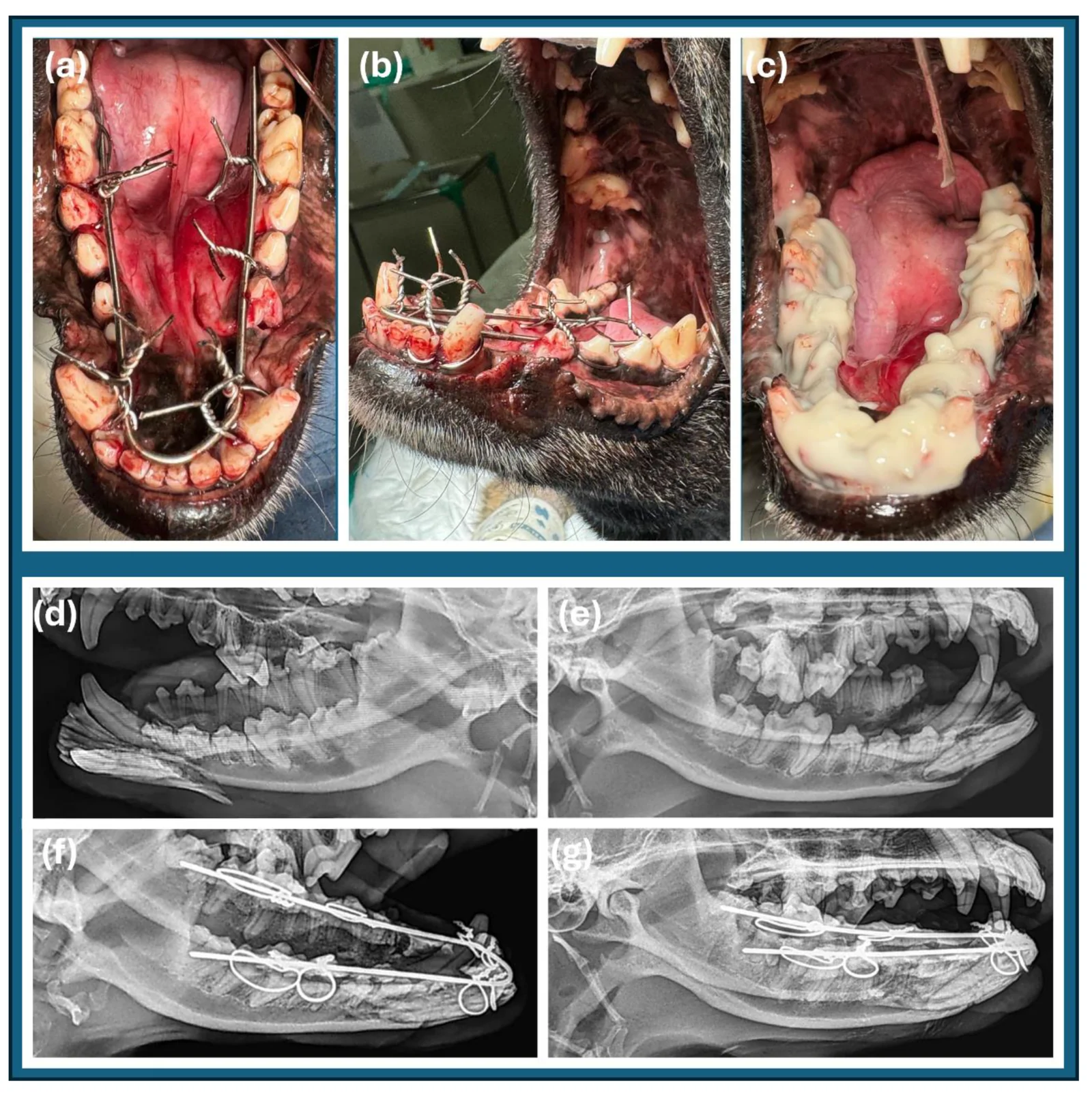
Photographic record (upper panel) and radiological study (lower panel) of Case 6. Lateral oblique projections (**d**,**e**) showed an alveolar fracture of the left first premolar (**d**) and the right canine (**e**). The incisive region was reduced and fixed using an intraoral lingual arch fixation device, with Type A anchors and bis-acrylic resin (**a**–**c**,**f**). Bone healing was complete in 63 days (**g**).

Case 6 (Figure 14)—a 5-year-old, intact female, 20.3 kg Belgian Shepherd dog—was presented 24 h after the onset of pain, bleeding, and difficulty closing the mouth as a result of being hit by a car. Two simple mandibular fractures, both open to the oral cavity, were diagnosed. The left mandible one was in the molar region and affected the left first premolar, which was luxated (alveolodental type I). The right fracture was in the incisive region, affecting the right canine (alveolodental type I), and the incisive region was displaced rostrally, causing malocclusion. The crowns of both canine teeth were worn, and the left second tooth presented a crown fracture with loss of its rostral third.

Fixation was performed using a 1.8 mm diameter lingual arch anchored to the first molar, third premolar, canine, and third incisor of the left mandible bone and to the third incisor, canine, fourth premolar, and first molar of the right mandibular bone using Type A anchors with a 0.8 mm diameter wire. The resin covered the bar, the cerclages, and the tooth necks of the mandibular jaw. After fixation, normocclusion was restored. Bone healing was completed 63 days after surgical treatment.

Bone healing was achieved in all cases (mean, 70.8 days; range, 63–96 days); after the radiological confirmation of bone healing, the intraoral fixator was removed. After removal of the intraoral fixator, with the patient sedated, the fracture site was manually assessed and confirmed to remain stable.

Occlusion and oral tissues were evaluated immediately and one-month post-removal. Normocclusion was maintained in all dogs. At the time of device removal, mild gingival irritation at the device contact points was observed in all cases and resolved within one week following topical chlorhexidine application. Within one month, normal oral function was confirmed, with no dental changes.

## 3. Discussion

The intraoral fixation technique described in this short case series resulted in bone healing of the fractures in all six patients, while restoring normocclusion and early oral function without causing any damage to the teeth, in accordance with the basic principles of mandibular fracture treatment. Although the intraoral fixation technique described in this study is based on the same basic components previously reported by Kern et al., it incorporates additional fixation methods, including Type B anchorage and circummandibular cerclage fixation, which distinguish the present technique from previously described systems and support its consideration as a novel surgical technique.

Providing adequate stability in the treatment of mandibular fractures is essential to achieve satisfactory bone healing within a clinically appropriate time frame [22,23]. It is even more important in unfavorable mandibular fractures, including simple fractures with an unfavorable directional configuration (dorsorostral to caudoventral orientation) as well as complex fractures [5,24]. In our case series, all the fracture configurations, except in Case 2, were unfavorable, in agreement with previously published reports [15]. As mentioned in the Introduction, our fixation system is based on the same components as the configuration that proved to be the most resistant in the biomechanical studies by Kern et al., being superior to the interdental cerclage and resin configuration. This finding is consistent with the results reported by Castejon et al. (2025), who described low strength and stiffness in intraoral splints associated with interdental cerclages [25]. We believe that our system could be more stable due to the presence of the arch-connecting bar that spans both mandibular bodies and because it features different types of connections between the teeth, the jaw, and the connecting bar. Nevertheless, we believe that it would be of great interest to conduct ex vivo biomechanical studies comparing this and other intraoral fixation systems with different configurations.

All patients had mandibular fractures that were open towards the oral cavity and potentially contaminated at the time of surgery. In these situations, the possibility of infection of internal implants and their explantation increases [26]; furthermore, the risk increases in cases with periodontal disease, as in Cases 1, 2, and 3. Plates require extensive approaches with periosteal detachment, which compromises local vascularization and facilitates bacterial colonization in contact with oral flora [20,21]. Interosseous cerclages, although less invasive, can cause local necrosis and intraoral exposure of the wires, increasing the possibility of infection and delayed healing [27] (Boudrieau, 2021). However, there were no infections observed in our case series associated with the use of the lingual arch intraoral device.

Interdental or interosseous cerclages, while simple and inexpensive, offer low stability and carry the risk of wire migration, malocclusion, infection, and delayed bone healing or nonunions [5,27]. For this reason, we believe that these techniques represent an excellent tool for achieving the reduction and primary fixation of anatomically reconstructible fractures, as in Cases 3 and 4. However, they should be combined with an additional system that neutralizes bending, rotational, and shear forces acting on the fractured mandible and therefore providing a protective function, as the lingual arch intraoral device described in this report does. Nevertheless, the successful fracture healing achieved in Cases 3 and 4 cannot be attributed exclusively to the intraoral fixation system, because a combination of fixation techniques was used in both cases.

The combined use of interdental cerclages and intraoral acrylic is a minimally invasive fixation technique that provides adequate fixation to allow bone healing while maintaining dental alignment and functional occlusion. Guzu and Hennet (2017) clinically evaluated this technique in dogs with mandibular fractures, showing a high rate of healing with minimal adverse effects [15]. However, this technique has certain limitations, one being the necessity of having healthy, properly positioned teeth (at least the first molar and the canine) on both sides of the fracture, a condition that may not be met in cases of pre-existing crown wear or tooth loss, either prior to the traumatic event or as a consequence of it. The use of Type B and C anchorages in our system presents an advantage in this regard, allowing its application in Case 3, in which one canine tooth and two incisors were absent and the remaining incisors showed worn crowns.

Case 2 was particularly noteworthy because the mandibular fracture was considered pathological due to the presence of stage IV periodontal disease, with substantial alveolar bone loss and multiple absent or mobile teeth. Following extraction of the mobile teeth, the limited number of remaining teeth reduced the available sites for dental anchorage. Nevertheless, the combination of Type A, Type Bf, and Type C anchorages allowed the fixation system to be adapted to the available teeth and mandibular bone. Despite these unfavorable local conditions, normal occlusion was restored after fixation and bone union with cortical bridging was achieved 96 days after treatment.

Because this fixation system relies on at least two anchors at each fracture site, we believe that it is ineffective for treating fractures caudally to the first molar, compared to other systems, where miniplates are more often indicated.

One of the main limitations of the present case series is the small sample size. Consequently, the results should be interpreted with caution and cannot be generalized to the broader population. Nevertheless, the favorable clinical outcomes observed in these cases suggest that this fixation technique may represent a promising alternative for the management of selected mandibular fractures. Further studies including larger case series are required to validate these preliminary findings. In addition, ex vivo biomechanical studies are warranted to objectively evaluate the mechanical stability of the fixation construct and determine whether it provides the level of stability suggested by the clinical outcomes observed in this study.

## 4. Conclusions

In the present case series, the application of the proposed mandibular fracture fixation system resulted in excellent clinical outcomes. The technique achieved successful bone healing within an appropriate healing period while restoring normal dental occlusion and without causing permanent damage to the teeth or to the mandibular neurovascular structures. These preliminary findings suggest that this fixation system represents a promising alternative for the management of selected mandibular fractures. However, given the limited number of cases included in this study, these results should be interpreted with caution. Further studies involving larger populations, together with ex vivo biomechanical investigations, are required to confirm the clinical effectiveness and mechanical stability of this fixation system.

## Figures and Tables

**Table 1:** Summary of the clinical characteristics and bone healing outcomes of the 11 mandibular body fractures treated in the six dogs included in the case series. Fracture complexity, location according to the alveolodental classification, communication with the oral cavity, type of bone healing, and time to bone healing are reported.

Number of Fracture	Case Number	Classification			Bone Healing	
		Complexity	Location	Communication	Type	Time (Days)
**1**	1	Simple	303–304 alveolodental type VI	Open towards oral cavity	Cortical bridging	64
**2**	1	Simple	404 alveolodental type I	Open towards oral cavity	Cortical bridging	64
**3**	2	Simple	404 alveolodental type I	Open towards oral cavity	Cortical bridging	96
**4**	3	Simple	304 alveolodental type I	Open towards oral cavity	Cortical bridging	67
**5**	3	Simple	404 alveolodental type II	Open towards oral cavity	Cortical bridging	67
**6**	4	Simple	307 alveolodental type III	Closed	Callus formation	71
**7**	4	Simple	308 alveolodental type VI	Open towards oral cavity	Callus formation	71
**8**	5	Simple	301–302 alveolodental type VI	Open towards oral cavity	Cortical bridging	64
**9**	5	Complex	404 alveolodental type I	Open towards oral cavity	Cortical bridging	64
**10**	6	Simple	305 alveolodental type I	Open towards oral cavity	Callus formation	63
**11**	6	Simple	404 alveolodental type I	Open towards oral cavity	Callus formation	63

## Data Availability

The data presented in this study are available on request from the corresponding author. The data are not publicly available due to privacy considerations related to client-owned animals. The original contributions presented in this study are included in the article. Further inquiries can be directed to the corresponding authors.
